# Striking Features of the Prosecution of the Atlanta Academy of Medicine

**Published:** 1871-05

**Authors:** 


### *Striking Features of the Prosecution of the Atlanta Academy of Medicine.*

Among the especially marked features of the late meeting of the Georgia Medical Association, were the two members from this city who acted in the capacity of *prosecutors* of the members of the Atlanta Academy of Medicine for associating with the late Faculty of the Atlanta Medical College, and as *exponents* of the Code of Ethics of the American Medical Association—one an expelled member of the same Faculty for gross and notorious violations of this Code, and the other a druggist of this city (not a practising physician), and daily violating this code by the extensive sale of patent nostrums. Truly we thought here is a most striking proof of the truth of the remark of some wise individual upon the cars as we went down, viz: "this is surely an age of progress."

These are the parties with whom the Savannah nobility affiliate in their work of upholding the "honor and dignity of the medical profession."

